# Supplementary material for: Genomic epidemiology of the 2025 mpox epidemic in Sierra Leone
Source: Nat Med. 2026 May 12;32(5):1917–26. doi: 10.1038/s41591-026-04385-8 (PMC13190292; doi:10.1038/s41591-026-04385-8)
Supplement: Supplementary file 1 — Reporting Summary [file 41591_2026_4385_MOESM1_ESM.pdf]

nature portfolio

Corresponding author(s): Allan K.O. Campbell, John Demby Sandi, Donald S. Grant

Last updated by author(s): Mar 31, 2026

Reporting Summary

Nature Portfolio wishes to improve the reproducibility of the work that we publish. This form provides structure for consistency and transparency in reporting. For further information on Nature Portfolio policies, see our Editorial Policies and the Editorial Policy Checklist.

Statistics

For all statistical analyses, confirm that the following items are present in the figure legend, table legend, main text, or Methods section.

n/a	Confirmed	
☐	☒	The exact sample size (n) for each experimental group/condition, given as a discrete number and unit of measurement
☐	☒	A statement on whether measurements were taken from distinct samples or whether the same sample was measured repeatedly
☒	☐	The statistical test(s) used AND whether they are one- or two-sided<br>*Only common tests should be described solely by name; describe more complex techniques in the Methods section.*
☐	☒	A description of all covariates tested
☒	☐	A description of any assumptions or corrections, such as tests of normality and adjustment for multiple comparisons
☐	☒	A full description of the statistical parameters including central tendency (e.g. means) or other basic estimates (e.g. regression coefficient) AND variation (e.g. standard deviation) or associated estimates of uncertainty (e.g. confidence intervals)
☒	☐	For null hypothesis testing, the test statistic (e.g. F, t, r) with confidence intervals, effect sizes, degrees of freedom and P value noted<br>*Give P values as exact values whenever suitable.*
☐	☒	For Bayesian analysis, information on the choice of priors and Markov chain Monte Carlo settings
☒	☐	For hierarchical and complex designs, identification of the appropriate level for tests and full reporting of outcomes
☒	☐	Estimates of effect sizes (e.g. Cohen's d, Pearson's r), indicating how they were calculated

Our web collection on statistics for biologists contains articles on many of the points above.

Software and code

Policy information about availability of computer code

Data collection	No software used for data collection
Data analysis	All code to run the analyses is available at GitHub (https://github.com/Ifeanyi-omah/Sierra_Leone_Mpox_project)<br>All software tools are listed in the Methods with version numbers: squirrel (O'Toole et al.), IQ-TREE v2.0, BEAST X v10.5.0, BEAGLE library (v3), TreeAnnotator 1.10, Tracer v1.7, LogCombiner, EpiFilter/Smooth, JUNIPER, BWA-MEM v0.7.17, SAMtools v1.6, iVar v1.3.1, Nextclade, R CODA package.

For manuscripts utilizing custom algorithms or software that are central to the research but not yet described in published literature, software must be made available to editors and reviewers. We strongly encourage code deposition in a community repository (e.g. GitHub). See the Nature Portfolio guidelines for submitting code & software for further information.

Data

Policy information about availability of data

All manuscripts must include a data availability statement. This statement should provide the following information, where applicable:

- Accession codes, unique identifiers, or web links for publicly available datasets
- A description of any restrictions on data availability
- For clinical datasets or third party data, please ensure that the statement adheres to our policy

All 338 MPXV genome sequences are deposited on Pathoplexus (https://doi.org/10.62599/PP_SS_232.1). Epidemiological case data are available at https://clt.npha.gov.sl/outbreak.aspx. Analysis code is available at https://github.com/Ifeanyi-omah/Sierra_Leone_Mpox_project. No other primary data were generated.

Research involving human participants, their data, or biological material

Policy information about studies with human participants or human data. See also policy information about sex, gender (identity/presentation), and sexual orientation and race, ethnicity and racism.

Reporting on sex and gender	Sex and age are collected via routine national surveillance.
Reporting on race, ethnicity, or other socially relevant groupings	Race and ethnicity are not routinely collected variables in Sierra Leone's public health surveillance system. Genomic data were anonymised prior to analysis.
Population characteristics	The study analysed n = 338 MPXV genomes from 338 individuals sampled across 14 districts in Sierra Leone, representing a geographically diverse population during the 2025 mpox outbreak.<br>Samples were obtained through routine national surveillance and outbreak response activities, encompassing both urban and rural districts. The distribution of samples reflects the national spread of mpox cases across multiple regions, as presented in the manuscript.<br>Demographic data, including age and sex, were collected as part of routine surveillance records. The affected population showed a near-equal sex distribution, with a slight skew toward younger women and older men, consistent with patterns described in the manuscript. The epidemic predominantly affected young adults (approximately 16–35 years).<br>All genomic data were anonymised prior to analysis. No sex-disaggregated genomic analyses were performed, as the study was designed as a population-level genomic investigation.
Recruitment	All confirmed mpox cases presenting to diagnostic facilities during the outbreak response were eligible. Samples were collected via the national surveillance system. Self-selection bias: cases from districts with better laboratory infrastructure (Western Area Urban, Kenema, Port Loko) are overrepresented; mild/asymptomatic cases not presenting to healthcare facilities are likely underrepresented.<br>All samples were collected between 10th January and 3rd August 2025 from outbreak response surveillance. The dataset was produced through collaborative efforts between the Central Public Health Reference Laboratory (CPHRL), Kenema Government Hospital (KGH) Viral Hemorrhagic Fever (VHF) Laboratory, and the Institut Pasteur de Dakar (IPD) mobile laboratory in Port-Loko. Samples were processed for DNA extraction at CPHRL and KGH VHF lab
Ethics oversight	This work was conducted as part of the public health response to the mpox outbreak under the mandate of the National Public Health Agency (NPHA), Ministry of Health, Sierra Leone, and all activities were performed in accordance with national outbreak investigation guidelines. As this investigation formed part of an emergency public health response, it was exempt from institutional ethics review and the requirement for individual informed consent under national public health regulations. All data were anonymized prior to analysis to protect patient confidentiality. All authors meet established authorship criteria and contributed substantially to study design, data generation, analysis, interpretation, and manuscript preparation. Local public health scientists and laboratory personnel were actively involved throughout the investigation and analysis, ensuring equitable collaboration, local ownership, and appropriate recognition of contributions. Care was taken to minimize stigma and harm in data handling, analysis, and reporting.

Note that full information on the approval of the study protocol must also be provided in the manuscript.

Field-specific reporting

Please select the one below that is the best fit for your research. If you are not sure, read the appropriate sections before making your selection.

☒ Life sciences ☐ Behavioural & social sciences ☐ Ecological, evolutionary & environmental sciences

For a reference copy of the document with all sections, see nature.com/documents/nr-reporting-summary-flat.pdf

Life sciences study design

All studies must disclose on these points even when the disclosure is negative.

Sample size	We generated 338 high-quality MPXV genomes from cases identified across the country between January 10 and August 3, 2025 (Figure 1A). The dataset was produced through collaboration among the Central Public Health Reference Laboratory (CPHRL, n=128 sequences), the Kenema Government Hospital (KGH) Viral Hemorrhagic Fever (VHF) Laboratory (n=105), and the Institut Pasteur de Dakar (IPD) mobile laboratory in Port-Loko (n=105). We also included two genomes from the Western Area Urban district sequenced by the Beijing Institute of Technology, bringing the total to 340 genomes<br>No formal power calculation was performed; this is a total-population genomic surveillance study where the sample size is determined by the epidemic itself, consistent with standard practice in genomic epidemiology.
Data exclusions	PCR-confirmed mpox cases with Ct <=30 and genome coverage >60% during the outbreak period. All qualifying samples from 10 Jan to 3 Aug 2025 were included (n=338)
Replication	Three chains of each Bayesian phylogenetic reconstruction was performed to assess whether they converge on the same posterior. They did. Uncertainty is quantified across the three combined independent chains, and across 10 000 phylogenetic trees in the posterior.This is an observational genomic epidemiology study with no experimental replication in the traditional sense. Phylogenetic and phylodynamic analyses were run as duplicate independent MCMC chains; both chains converged (ESS >200) and results were consistent. JUNIPER was run across two chains with consistent posterior distributions
Randomization	Irrelevant as there are no experimental groups.
Blinding	Irrelevant as metadata is vital to analyses.

Reporting for specific materials, systems and methods

We require information from authors about some types of materials, experimental systems and methods used in many studies. Here, indicate whether each material, system or method listed is relevant to your study. If you are not sure if a list item applies to your research, read the appropriate section before selecting a response.

Materials & experimental systems

n/a	Involved in the study	
☒	☐	Antibodies
☒	☐	Eukaryotic cell lines
☒	☐	Palaeontology and archaeology
☒	☐	Animals and other organisms
☒	☐	Clinical data
☒	☐	Dual use research of concern
☒	☐	Plants

Methods

n/a	Involved in the study	
☒	☐	ChIP-seq
☒	☐	Flow cytometry
☒	☐	MRI-based neuroimaging

Plants

Seed stocks	*Report on the source of all seed stocks or other plant material used. If applicable, state the seed stock centre and catalogue number. If plant specimens were collected from the field, describe the collection location, date and sampling procedures.*
Novel plant genotypes	*Describe the methods by which all novel plant genotypes were produced. This includes those generated by transgenic approaches, gene editing, chemical/radiation-based mutagenesis and hybridization. For transgenic lines, describe the transformation method, the number of independent lines analyzed and the generation upon which experiments were performed. For gene-edited lines, describe the editor used, the endogenous sequence targeted for editing, the targeting guide RNA sequence (if applicable) and how the editor was applied.*
Authentication	*Describe any authentication procedures for each seed stock used or novel genotype generated. Describe any experiments used to assess the effect of a mutation and, where applicable, how potential secondary effects (e.g. second site T-DNA insertions, mosiacism, off-target gene editing) were examined.*
